# 免疫单药与免疫联合化疗在75岁及以上晚期非小细胞肺癌患者中的疗效与安全性对比

**DOI:** 10.3779/j.issn.1009-3419.2024.101.21

**Published:** 2024-09-20

**Authors:** Yunye MAO, An WANG, Shu SHENG, Yangyang JIA, Xiangwei GE, Jinzhao ZHAI, Jinliang WANG

**Affiliations:** ^1^100071 北京，解放军总医院第五医学中心肿瘤医学部肿瘤内科; ^1^Department of Oncology, Senior Department of Oncology, the Fifth Medical Center of PLA General Hospital, Beijing 100071, China; ^2^100853 北京，解放军医学院; ^2^Chinese PLA Medical School, Beijing 100853, China

**Keywords:** 肺肿瘤, 老年患者, 免疫治疗, Lung neoplasms, The elderly patients, Immunotherapy

## Abstract

**背景与目的:**

肺癌是全球范围内发病率和死亡率最高的恶性肿瘤，且多发生于老年患者，免疫检查点抑制剂（immune checkpoint inhibitors, ICIs）的应用改变了非小细胞肺癌（non-small cell lung cancer, NSCLC）的治疗格局，本研究旨在评估75岁及以上晚期NSCLC患者接受免疫单药与免疫联合化疗治疗的疗效与安全性。

**方法:**

本研究采用回顾性分析，纳入2018年1月至2022年10月在解放军总医院第一、第五医学中心接受治疗的111例75岁及以上的晚期NSCLC患者。这些患者接受了一线或二线治疗，其中70例接受免疫联合化疗，41例接受免疫单药治疗。采用倾向性评分匹配（propensity score matching, PSM）法对患者的基线特征进行匹配，包括年龄、东部肿瘤协作组体力状态（Eastern Cooperative Oncology Group performance status, ECOG PS）评分和治疗线数。研究终点包括客观缓解率（objective response rate, ORR）、无进展生存期（progression-free survival, PFS）、总生存期（overall survival, OS）以及药物的安全性。

**结果:**

免疫联合化疗组的中位OS为27.87个月，中位PFS为11.50个月，免疫单药组的中位OS为34.93个月，中位PFS为17.00个月，两者的OS（P=0.722）和PFS（P=0.474）无明显差异，ORR之间可见统计学差异（P=0.025）。PSM匹配后，两组各27例患者，免疫联合化疗组的中位OS为17.70个月，中位PFS为8.97个月；免疫单药组的中位OS为17.87个月，中位PFS为11.53个月，两组间OS（P=0.635）和PFS（P=0.878）均无统计学差异，ORR也未观察到统计学差异（P=0.097）。安全性方面，匹配前免疫联合化疗组总体不良反应（adverse events, AEs）发生率为62.86%，高于免疫单药组的41.46%（P=0.029），而≥3级AEs发生率在两组间无差异（P=0.221）。匹配后，免疫联合化疗组17例（62.96%）患者中有AEs发生，免疫单药组有13例（48.15%），二组不论是总体AEs发生率（P=0.273）还是≥3级AEs发生率（P=0.299）均未见明显差异。

**结论:**

在75岁及以上的晚期NSCLC患者中，免疫联合化疗相较于免疫单药治疗，并未提供显著的OS或PFS优势，PSM后的分析进一步验证了这一结论。安全性评估表明，匹配前免疫联合化疗组任意等级AEs的发生率高，但≥3级AEs发生率两组无差异，匹配后两组治疗的耐受性相似。安全性评估提示在为老年晚期NSCLC患者制定治疗方案时，应综合考虑患者的具体情况和治疗风险。

## Abstract (en)

**Background and objective:**

The malignant tumor that has the highest global morbidity and death rate is lung cancer, which primarily affects the elderly. The therapy landscape for non-small cell lung cancer (NSCLC) has transformed with the introduction of immune checkpoint inhibitors (ICIs). The purpose of this study was to compare the safety and efficacy of immune monotherapy and immunotheray combined with chemotherapy in patients with advanced NSCLC aged 75 years and above.

**Methods:**

This study retrospectively analyzed 111 patients with advanced NSCLC who were at least 75 years old and received treatment at the First or Fifth Medical Centers of the People's Liberation Army General Hospital from January 2018 to October 2022. These patients underwent first-line or second-line treatment, with 70 receiving immunotherapy combined with chemotherapy and 41 receiving immunotherapy alone. Propensity score matching (PSM) was used to match the baseline characteristics of the patients, including age, Eastern Cooperative Oncology Group performance status (ECOG PS) score, and the number of treatment lines. The study endpoints included objective response rate (ORR), progression-free survival (PFS), overall survival (OS), and safety assessment.

**Results:**

The median OS for the immunotherapy combined with chemotherapy group was 27.87 months, and the median PFS was 11.50 months. The median OS for the immune monotherapy group was 34.93 months, and the median PFS was 17.00 months. There were no significant differences in OS (P=0.722) and PFS (P=0.474) between the two groups, but a significant difference was observed in ORR (P=0.025). After PSM matching, each group comprised 27 patients. The median OS for the immunotherapy combined with chemotherapy group was 17.70 months, the median PFS was 8.97 months. The median OS for the immune monotherapy group was 17.87 months, and the median PFS was 11.53 months. No significant differences were observed in OS (P=0.635), PFS (P=0.878) and ORR (P=0.097). In terms of safety, the overall incidence of adverse events (AEs) before matching was 62.86% in the immunotherapy combined with chemotherapy group, which was higher than 41.46% in the immune monotherapy group (P=0.029), while there was no difference in the incidence of AEs of grade 3 or above between the two groups (P=0.221). After matching, AEs occurred in 17 (62.96%) patients in the immunotherapy combined with chemotherapy group and 13 (48.15%) in the immune monotherapy group. There were no significant differences in the overall incidence of AEs (P=0.273) or the incidence of grade 3 or above (P=0.299) between the two groups.

**Conclusion:**

Immunotherapy combined with chemotherapy does not significantly improve OS or PFS in patients with NSCLC aged 75 years and above when compared to immunotherapy alone, and this conclusion was further validated by the analysis after PSM. The safety assessment suggests that before matching, the incidence of AEs of any grade in the immunotherapy combined with chemotherapy group was higher. Still, the two groups had no difference in the incidence of AEs of grade 3 or above. Following matching, the tolerability of the treatment was similar in both groups. According to the safety assessment, the unique circumstances and course of treatment for geriatric patients with advanced NSCLC should be considered.

数据^[1,2]^显示，全球新发肺癌248万（12.4%），这是肺癌在2020年被乳腺癌超越后，再次成为全球第一大癌症，死亡180万（18.7%），仍居癌症死亡首位。其中约50%的肺癌全球新发及死亡病例发生在亚洲，而我国肺癌仍是癌症相关新发病率和病死率的首要原因，且呈增高趋势。肺癌发病风险随着年龄的增长而增加，我国国家癌症中心数据^[3]^显示，40岁后肺癌的发病率逐渐上升，在80岁达峰值。非小细胞肺癌（non-small cell lung cancer, NSCLC）占所有肺癌的80%以上，约一半确诊时超过70岁，大多数早期肺癌患者常无症状，在诊断时已是晚期，且5年生存率低于15%^[4]^。

然而，随着免疫治疗的兴起，特别是免疫检查点抑制剂（immune checkpoint inhibitors, ICIs）的应用，NSCLC的治疗格局发生了显著变化，ICIs已成为驱动基因阴性NSCLC治疗的标准。多项大型研究^[5,6]^表明，无论细胞程序性死亡配体1（programmed cell death ligand 1, PD-L1）表达水平如何，免疫联合化疗均能为晚期NSCLC患者带来显著临床获益，且安全性可控。然而，老年患者，尤其是75岁及以上患者，由于身体状况差等原因，在各项实验中的代表性不足^[7]^。因此这部分患者是否能从免疫联合化疗中获益，以及其安全性如何，尚需进一步研究。本研究通过回顾性分析旨在评估75岁及以上晚期NSCLC患者接受免疫单药治疗或免疫联合治疗的疗效和安全性，以期为老年NSCLC患者，特别是75岁及以上患者的临床治疗提供更多依据。

## 1 资料与方法

### 1.1 一般资料

回顾性收集2018年1月至2022年10月就诊于解放军总医院第一、第五医学中心的晚期NSCLC患者的临床资料。纳入标准：（1）病理类型为NSCLC，临床分期按照美国癌症联合委员会（American Joint Committee on Cancer, AJCC）第九版肺癌分期标准为IIIB-IV期；（2）年龄≥75岁；（3）驱动基因突变阴性；（4）一线或二线接受免疫治疗；（5）基线资料完整，按照实体瘤疗效评价标准（Response Evaluation Criteria in Solid Tumors, RECIST）1.1版，至少有一个可测量评估病灶。排除标准：（1）多部位原发肿瘤；（2）临床资料不完整；（3）治疗少于2个周期。最后共纳入111例患者。本研究通过解放军总医院临床研究伦理审批（伦理号：2023-8-26-1），获得全部研究参与者的知情同意。

### 1.2 资料收集

查阅解放军总医院第一、第五医学中心的电子病历，采集患者基线信息与治疗情况，包括：性别、年龄、吸烟史、东部肿瘤协作组体力状态（Eastern Cooperative Oncology Group performance status, ECOG PS）评分、PD-L1表达、肿瘤类型、肿瘤分期、转移情况、免疫治疗情况、不良反应（adverse events, AEs）等。

### 1.3 疗效评价

根据RECIST 1.1进行评估，分为完全缓解（complete response, CR）、部分缓解（partial response, PR）、疾病稳定（stable disease, SD）和疾病进展（progressive disease, PD）。主要研究终点是总生存期（overall survival, OS），定义为自接受免疫治疗起至死亡的时间。次要终点为无进展生存期（progression-free survival, PFS），定义为自接受免疫治疗起至疾病复发、进展或死亡的时间。客观缓解率（objective response rate, ORR）为（CR+PR）例数/总例数×100%。研究截止2023年9月30日未达到终点的事件按截尾数据处理。AEs按照不良事件通用评价标准（Common Terminology Criteria for Adverse Events, CTCAEs）5.0进行评估。

### 1.4 统计学处理

采用SPSS 26.0软件进行统计学分析，采用1:1倾向性评分匹配（propensity score matching, PSM）均衡基线信息，卡钳值设定为0.05。使用Kaplan-Meier方法绘制生存曲线，Log-rank检验进行组间比较，计量资料比较采用t检验，不符合正态分布的数据采用秩和检验。计数资料采用χ^2^检验，使用Cox回归风险比例模型进行单因素、多因素分析，双侧P<0.05表示差异有统计学意义。

## 2 结果

### 2.1 一般资料

本研究共纳入111例75岁及以上的晚期NSCLC患者，其中41例接受免疫单药治疗，70例则接受免疫联合化疗。采用PSM法均衡患者的基线特征，包括年龄、ECOG PS评分和治疗线数，匹配后两组基线信息无统计学差异（P>0.05）。两组患者PSM前后的临床特征见表1。

**表1:** 患者基线信息

Variables	Before PSM After PSM									
	Total (n=111)	Monotherapy (n=41)	Combination (n=70)	P			Total (n=54)	Monotherapy (n=27)	Combination (n=27)	P
Age (yr), Median (range)	78 (75-91)	79 (75-91)	77 (75-87)	0.007			79 (75-86)	78 (75-86)	79 (75-86)	0.773
Gender, n (%)				0.610						>0.999
Male	95 (85.59)	36 (87.81)	59 (84.29)				44 (81.48)	22 (81.48)	22 (81.48)	
Female	16 (14.41)	5 (12.19)	11 (15.71)				10 (18.52)	5 (18.52)	5 (18.52)	
ECOG PS, n (%)				0.022						0.299
0	7 (6.31)	2 (4.88)	5 (7.14)				1 (1.85)	1 (3.70)	0 (0.00)	
1	88 (79.28)	28 (68.29)	60 (85.72)				46 (85.19)	21 (77.78)	25 (92.59)	
2	14 (12.61)	9 (21.95)	5 (7.14)				6 (11.11)	4 (14.82)	2 (7.41)	
3	2 (1.80)	2 (4.88)	0 (0.00)				1 (1.85)	1 (3.70)	0 (0.00)	
Smoking status, n (%)				0.449						0.475
Never	36 (32.43)	13 (31.71)	23 (32.86)				17 (31.48)	8 (29.63)	9 (33.33)	
Former	47 (42.34)	15 (36.58)	32 (45.71)				21 (38.89)	9 (33.33)	12 (44.45)	
Current	28 (25.23)	13 (31.71)	15 (21.43)				16 (29.63)	10 (37.04)	6 (22.22)	
Pathology, n (%)				0.817						0.457
Squamous	46 (41.44)	16 (39.02)	30 (42.86)				24 (44.44)	11 (40.74)	13 (48.15)	
Adenocarcinoma	58 (52.25)	23 (56.10)	35 (50.00)				26 (48.15)	15 (55.56)	11 (40.74)	
Other	7 (6.31)	2 (4.88)	5 (7.14)				4 (7.41)	1 (3.70)	3 (11.11)	
T stage, n (%)				0.087						0.764
1	17 (15.31)	5 (12.19)	12 (17.14)				4 (7.41)	2 (7.41)	2 (7.41)	
2	53 (47.75)	23 (56.10)	30 (42.86)				26 (48.15)	14 (51.85)	12 (44.44)	
3	28 (25.23)	12 (29.27)	16 (22.86)				20 (37.03)	10 (37.04)	10 (37.04)	
4	13 (11.71)	1 (2.44)	12 (17.14)				4 (7.41)	1 (3.70)	3 (11.11)	
N stage, n (%)				0.246						0.434
0	16 (14.41)	8 (19.51)	8 (11.43)				10 (18.52)	5 (18.52)	5 (18.52)	
1	8 (7.21)	3 (7.32)	5 (7.14)				5 (9.26)	2 (7.41)	3 (11.11)	
2	62 (55.86)	18 (43.90)	44 (62.86)				26 (48.15)	11 (40.74)	15 (55.56)	
3	25 (22.52)	12 (29.27)	13 (18.57)				13 (24.07)	9 (33.33)	4 (14.81)	
Clinical stage, n (%)				0.812						0.214
III	34 (30.63)	12 (29.27)	22 (31.43)				14 (25.93)	9 (33.33)	5 (18.52)	
IV	77 (69.37)	29 (70.73)	48 (68.57)				40 (74.07)	18 (66.67)	22 (81.48)	
Liver metastasis, n (%)				0.679						>0.999
No	103 (92.79)	37 (90.24)	66 (94.29)				51 (94.44)	25 (92.59)	26 (96.30)	
Yes	8 (7.21)	4 (9.76)	4 (5.71)				3 (5.56)	2 (7.41)	1 (3.70)	
Bone metastasis, n (%)				0.766						0.362
No	83 (74.77)	30 (73.17)	53 (75.71)				39 (72.22)	21 (77.78)	18 (66.67)	
Yes	28 (25.23)	11 (26.83)	17 (24.29)				15 (27.78)	6 (22.22)	9 (33.33)	
Intrapulmonary metastasis, n (%)				0.355						0.535
No	89 (80.18)	31 (75.61)	58 (82.86)				40 (74.07)	19 (70.37)	21 (77.78)	
Yes	22 (19.82)	10 (24.39)	12 (17.14)				14 (25.93)	8 (29.63)	6 (22.22)	
Brain metastasis, n (%)				0.533						>0.999
No	105 (94.59)	40 (97.56)	65 (92.86)				51 (94.44)	26 (96.30)	25 (92.59)	
Yes	6 (5.41)	1 (2.44)	5 (7.14)				3 (5.56)	1 (3.70)	2 (7.41)	
Pleural effusion, n (%)				0.971						0.551
No	81 (72.97)	30 (73.17)	51 (72.86)				38 (70.37)	20 (74.07)	18 (66.67)	
Yes	30 (27.03)	11 (26.83)	19 (27.14)				16 (29.63)	7 (25.93)	9 (33.33)	
Other metastasis, n (%)				0.578						0.551
No	74 (66.67)	26 (63.41)	48 (68.57)				38 (70.37)	18 (66.67)	20 (74.07)	
Yes	37 (33.33)	15 (36.59)	22 (31.43)				16 (29.63)	9 (33.33)	7 (25.93)	
Complication, n (%)				0.820						0.785
No	53 (47.75)	19 (46.34)	34 (48.57)				25 (46.30)	13 (48.15)	12 (44.44)	
Yes	58 (52.25)	22 (53.66)	36 (51.43)				29 (53.70)	14 (51.85)	15 (55.56)	
Local treatment, n (%)				0.115						0.362
No	85 (76.58)	28 (68.29)	57 (81.43)				39 (72.22)	18(66.67)	21(77.78)	
Yes	26 (23.42)	13 (31.71)	13 (18.57)				15 (27.78)	9(33.33)	6(22.22)	
Treatment line, n (%)				0.009						0.735
1^st^	92 (82.88)	29 (70.73)	63 (90.00)				43 (79.63)	22 (81.48)	21 (77.78)	
2^nd^	19 (17.12)	12 (29.27)	7 (10.00)				11 (20.37)	5 (18.52)	6 (22.22)	
PD-L1, n (%)				0.649						0.402
<1%	18 (16.22)	6(14.63)	12 (17.14)				7 (12.96)	4 (14.81)	3 (11.11)	
1%-49%	36 (32.43)	13 (31.71)	23 (32.86)				16 (29.63)	8 (29.63)	8 (29.63)	
≥50%	18 (16.22)	9 (21.95)	9 (12.86)				10 (18.52)	7 (25.93)	3 (11.11)	
Unknown	39 (35.13)	13 (31.71)	26 (37.14)				21 (38.89)	8 (29.63)	13 (48.15)	

PSM: propensity score matching; ECOG PS: Eastern Cooperative Oncology Group performance status; PD-L1: programmed cell death ligand 1.

### 2.2 疗效分析

在随访结束时，免疫单药组和免疫联合化疗组分别有17例（41.46%）和31例（44.29%）经历了总生存事件。在疗效评估方面，免疫单药组中，有10例（24.39%）实现PR，25例（60.98%）SD，6例（14.63%）PD。而在免疫联合化疗组中，32例（45.71%）达到PR，36例（51.43%）SD，2例（7.41%）PD。单药组ORR为24.39%，而联合化疗组ORR为45.71%，两组间可见显著差异（P=0.025）（表2）。生存期分析方面，免疫联合化疗组的中位OS达到27.87（95%CI: 16.41-39.33）个月，中位PFS为11.50（95%CI: 6.26-16.74）个月，而免疫单药组的中位OS为34.93（95%CI: NA-NA）个月，中位PFS为17.00（95%CI: 8.51-25.49）个月。与免疫单药相比，免疫联合化疗既没有提高患者的OS（HR=1.11, 95%CI: 0.62-2.02, P=0.722），也没有提高患者的PFS（HR=1.19, 95%CI: 0.74-1.89, P=0.474）（图1A、1B）。

**表2:** PSM前后两组患者的临床疗效比较

Items	CR	PR	SD	PD	ORR	P
Before PSM						0.025
Monotherapy (n=41)	0 (0.00%)	10 (24.39%)	25 (60.98%)	6 (14.63%)	10 (24.39%)	
Combination (n=70)	0 (0.00%)	32 (45.71%)	36 (51.43%)	2 (2.86%)	32 (45.71%)	
After PSM						0.097
Monotherapy (n=27)	0 (0.00%)	8 (29.63%)	15 (55.56%)	4 (14.81%)	8 (29.63%)	
Combination (n=27)	0 (0.00%)	14 (51.85%)	11 (40.74%)	2 (7.41%)	14 (51.85%)	

CR: complete response; PR: partial response; SD: stable disease; PD: progressive disease; ORR: objective response rate.

**图1:**
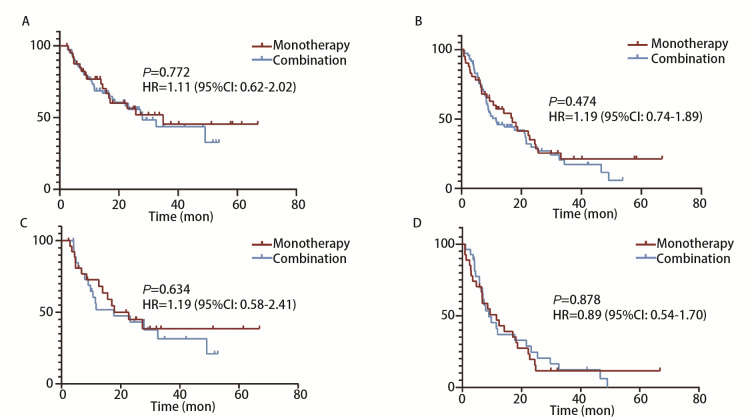
Kaplan-Meier曲线。A：PSM前患者的OS；B：PSM前患者的PFS；C：PSM后患者的OS；D：PSM后患者的PFS。

采用PSM法均衡两组患者特征后，每组纳入27例患者。随访截止时，免疫单药组中有14例（51.85%）患者发生了总生存事件，而免疫联合化疗组有17例（62.96%）。在疗效评估方面，免疫单药组与免疫联合化疗组分别有8例（29.63%）和14例（51.85%）患者疗效为PR，15例（55.56%）和11例（40.74%）患者疗效为SD，4例（14.81%）和2例（7.41%）患者疗效为PD。两组患者的ORR分别为29.63%和51.85%，未见统计学差异（P=0.097）（表2）。免疫联合化疗组的中位OS为17.70（95%CI: 9.19-35.71）个月，中位PFS为8.97（95%CI: 4.06-13.88）个月，免疫单药组则分别为17.87（95%CI: 7.62-28.12）个月和11.53（95%CI: 4.55-18.51）个月。两组在OS（HR=1.19, 95%CI: 0.58-2.41, P=0.635）和PFS（HR=0.88, 95%CI: 0.54-1.70, P=0.878）方面均未发现显著差异（图1C、1D）。

单因素分析表明，处于肿瘤原发灶-淋巴结-转移（tumor-node-metastasis, TNM）分期N2期的患者（HR=4.77, 95%CI: 1.01-20.73, P=0.037）以及存在骨转移的患者（HR=2.22, 95%CI: 1.07-4.59, P=0.032）有更高的死亡风险。相对的，接受局部治疗（HR=0.29, 95%CI: 0.10-0.84, P=0.022）及PD-L1表达在1%-49%（HR=0.20, 95%CI: 0.06-0.67, P=0.009）的患者死亡风险降低。进一步多因素回归分析显示，接受局部治疗（HR=0.20, 95%CI: 0.07-0.62, P=0.005）和PD-L1表达在1%-49%（HR=0.22, 95%CI: 0.07-0.74, P=0.015）是与OS的获益显著相关的预后因素（表3）。此外，亚组分析中可以看出，IIIB期患者使用免疫联合化疗疗效更佳（P=0.029），其他因素在疗效上未见明显差异（表4）。

**表3:** OS的Cox回归分析

Variables	Univariate analysis			Multivariate analysis	
	P	HR (95%CI)		P	HR (95%CI)
Age	0.643	1.03 (0.91-1.16)			
Gender					
Male					
Female	0.716	0.85 (0.35-2.07)			
ECOG PS					
0-1					
≥2	0.278	1.71 (0.65-4.51)			
Smoking status					
Never					
Former	0.627	1.23 (0.53-2.85)			
Current	0.847	1.10 (0.42-2.86)			
Pathology					
Squamous					
Adenocarcinoma	0.397	0.72 (0.34-1.53)			
Other	0.155	2.61 (0.70-9.77)			
T stage					
1					
2	0.605	1.71 (0.22-13.20)			
3	0.215	3.62 (0.48-27.61)			
4	0.591	1.87 (0.19-18.13)			
N stage					
0					
1	0.228	3.01 (0.50-18.12)			
2	0.037	4.77 (1.10-20.73)			
3	0.088	3.81 (0.82-17.70)			
Clinical stage					
III					
IV	0.068	2.45 (0.94-6.41)			
Liver metastasis					
No					
Yes	0.097	3.52 (0.80-15.56)			
Bone metastasis					
No					
Yes	0.032	2.22 (1.07-4.59)			
Intrapulmonary metastasis					
No					
Yes	0.460	1.36 (0.60-3.05)			
Brain metastasis					
No					
Yes	0.443	0.46 (0.06-3.37)			
Pleural effusion					
No					
Yes	0.123	1.81 (0.85-3.82)			
Other metastasis					
No					
Yes	0.083	1.92 (0.92-4.03)			
Complication					
No					
Yes	0.169	1.68 (0.80-3.52)			
Local treatment					
No					
Yes	0.022	0.29 (0.10-0.84)		0.005	0.20 (0.07-0.62)
Treatment line					
1^st^					
2^nd^	0.507	0.74 (0.30-1.82)			
Adverse events					
No					
Yes	0.626	0.84 (0.41-1.70)			
Grade 1-2					
No					
Yes	0.988	1.01 (0.49-2.06)			
Grade 3-4					
No					
Yes	0.418	0.55 (0.13-2.32)			
PD-L1					
<1%					
1%-49%	0.009	0.20 (0.06-0.67)		0.015	0.22 (0.07-0.74)

**表4:** 患者OS的亚组分析

Subgroup	n (%)	HR (95%CI)	P
All patients	54 (100.00)	1.19 (0.58-2.41)	0.636
Gender			
Male	44 (81.48)	1.49 (0.67-3.31)	0.333
Female	10 (18.52)	0.47 (0.09-2.58)	0.384
ECOG PS			
0-1	47 (87.04)	1.22 (0.56-2.67)	0.612
≥2	7 (12.96)	0.66 (0.07-6.74)	0.729
Smoking status			
Never	17 (31.48)	0.79 (0.21-2.97)	0.732
Former/Current	37 (68.52)	1.54 (0.66-3.62)	0.319
Pathology			
Squamous	24 (44.44)	1.38 (0.45-4.25)	0.575
Non-squamous	30 (55.56)	1.03 (0.41-2.60)	0.951
Clinical stage			
III	14 (25.93)	11.73 (1.28-107.30)	0.029
IV	40 (74.07)	0.60 (0.28-1.30)	0.197
Liver metastasis			
No	51 (94.44)	1.40 (0.67-2.94)	0.373
Yes	3 (5.56)	0.00 (0.00-Inf)	0.999
Bone metastasis			
No	39 (72.22)	1.48 (0.60-3.65)	0.395
Yes	15 (27.78)	0.33 (0.09-1.25)	0.102
Intrapulmonary metastasis			
No	40 (74.07)	1.53 (0.66-3.53)	0.324
Yes	14 (25.93)	0.70 (0.17-2.98)	0.634
Brain metastasis			
No	51 (94.44)	1.26 (0.61-2.59)	0.531
Yes	3 (5.56)	NA	NA
Pleural effusion			
No	38 (70.37)	1.48 (0.61-3.59)	0.390
Yes	16 (29.63)	0.50 (0.15-1.66)	0.255
Other metastasis			
No	38 (70.37)	1.30 (0.53-3.20)	0.563
Yes	16 (29.63)	1.19 (0.36-3.93)	0.773
Complication			
No	25 (46.30)	0.76 (0.23-2.51)	0.655
Yes	29 (53.70)	1.58 (0.64-3.89)	0.320
Treatment line			
1^st^	43 (79.63)	1.72 (0.78-3.80)	0.182
2^nd^	11 (20.37)	0.41 (0.08-2.07)	0.279
Adverse events			
No	24 (44.44)	0.89 (0.33-2.38)	0.809
Yes	30 (55.56)	1.66 (0.56-4.88)	0.357

NA: not available.

### 2.3 安全性

在对匹配前患者进行安全性评估时，发现在免疫联合化疗组中，发生任意等级AEs为44例（62.86%），其中10例（14.29%）患者出现≥3级的AEs，主要为骨髓抑制（12.86%）、肝毒性（1.43%）和肾毒性（1.43%）。相比之下，免疫单药组中发生任意等级AEs为17例（41.46%），较为常见的包括6例（14.63%）骨髓抑制、3例（7.32%）胃肠道反应和3例（7.32%）皮疹等，2例（4.88%）患者出现≥3级AEs，均为骨髓抑制（表5）。两组相比发现，免疫联合化疗组任意等级AEs的发生率高（P=0.029），但≥3级AEs发生率两组无差异（P=0.221）（图2A）。匹配后，免疫单药组中有13例（48.15%）患者出现AEs，其中1例（3.70%）患者出现≥3级AEs。免疫联合化疗组中则有17例（62.96%）患者出现AEs，其中3例（11.11%）患者出现≥3级AEs（表5）。两组间无论是任意等级AEs（P=0.273）还是≥3级AEs的发生率（P=0.299）均未见统计学差异（图2B）。

**表5:** PSM前后具体不良反应

Adverse events	Before PSM				After PSM			
	Monotherapy (n=41)		Combination (n=70)		Monotherapy (n=27)		Combination (n=27)	
	Any grade	Grade≥3	Any grade	Grade≥3	Any grade	Grade≥3	Any grade	Grade≥3
Any	17 (41.46%)	2 (4.88%)	44 (62.86%)	10 (14.29%)	13 (48.15%)	1 (3.70%)	17 (62.96%)	3 (11.11%)
Myelosuppression	6 (14.63%)	2 (4.88%)	22 (31.43%)	9 (12.86%)	5 (18.52%)	1 (3.70%)	9 (33.33%)	3 (11.11%)
Gastrointestinal response	3 (7.32%)	0 (0.00%)	14 (20.00%)	0 (0.00%)	3 (11.11%)	0 (0.00%)	4 (14.81%)	0 (0.00%)
Hepatotoxicity	2 (4.88%)	0 (0.00%)	12 (17.14%)	1 (1.43%)	2 (7.41%)	0 (0.00%)	6 (22.22%)	0 (0.00%)
Nephrotoxicity	2 (4.88%)	0 (0.00%)	4 (5.71%)	1 (1.43%)	1 (3.70%)	0 (0.00%)	0 (0.00%)	0 (0.00%)
Immune-related pneumonia	2 (4.88%)	0 (0.00%)	2 (2.86%)	0 (0.00%)	2 (7.41%)	0 (0.00%)	2 (7.41%)	0 (0.00%)
Rash	3 (7.32%)	0 (0.00%)	4 (5.71%)	0 (0.00%)	1 (3.70%)	0 (0.00%)	1 (3.70%)	0 (0.00%)
Hypothyroidism	2 (4.88%)	0 (0.00%)	1 (1.43%)	0 (0.00%)	2 (7.41%)	0 (0.00%)	0 (0.00%)	0 (0.00%)
Others	11 (26.83%)	0 (0.00%)	12 (17.14%)	0 (0.00%)	6 (22.22%)	0 (0.00%)	5 (18.52%)	0 (0.00%)

**图2:**
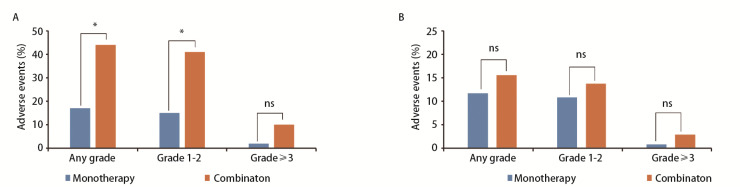
PSM前（A）、后（B）的不良反应

## 3 讨论

肺癌作为全球范围内发病率和死亡率最高的恶性肿瘤，对人类健康构成了巨大威胁。近年来，ICIs已成为NSCLC治疗领域的重要突破，通过调节肿瘤微环境中的免疫反应，显著提升了患者的生存率和生活质量^[8]^。全球多中心III期临床研究KEYNOTE-189^[9]^和KEYNOTE-407^[10]^表明，免疫联合化疗在晚期非鳞状和鳞状NSCLC患者中的一线治疗相比单纯化疗具有显著的疗效优势，从而确立了免疫联合化疗在晚期驱动基因阴性NSCLC治疗中的标准地位。

在疗效方面，本研究发现无论是匹配前还是匹配后的患者，免疫单药与免疫联合治疗之间无疗效差异。同样在一项为评估免疫联合化疗对既往未经治疗的老年晚期NSCLC患者的安全性和有效性的回顾性研究^[11]^中发现，对于75岁及以上的患者，与单独使用免疫治疗相比，免疫联合化疗并不能提高生存率。一项发表在美国肿瘤临床学会（American Society of Clinical Oncology, ASCO）会议上的摘要表明，无论PD-L1表达在1%-49%或≥50%，75岁及以上的NSCLC患者免疫联合化疗的疗效并没有优于免疫单药^[12,13]^。另外一项汇总KEYNOTE-010、KEYNOTE-024和KEYNOTE-042的研究^[14]^共纳入2612例接受免疫单药对比化疗治疗的晚期NSCLC患者，其中75岁及以上患者264例，中位随访时间为11.7个月，研究发现免疫单药组和化疗组的中位OS分别为15.7和11.7个月（HR=0.76, 95%CI: 0.56-1.02），这与我们匹配后患者免疫单药中位OS基本一致。但现有大部分研究未能充分纳入老年患者群体，特别是年龄在75岁及以上的患者。考虑到这一患者群体的特殊需求和治疗挑战，本研究通过收集和分析真实世界数据，旨在对比评估免疫单药治疗与免疫联合化疗在75岁及以上晚期NSCLC患者中的疗效与安全性。这项研究为这一特定患者群体提供了新的治疗依据，有助于指导临床决策，改善老年晚期NSCLC患者的治疗策略。

在安全性方面，匹配前数据显示免疫联合化疗组的任意等级AEs发生率较高，但在严重的AEs（≥3级）方面，两组之间并没有统计学差异。经过PSM消除基线差异后，我们的研究并未发现免疫联合治疗与免疫单药治疗在AEs发生率方面有显著差异，75岁及以上的患者用药总体安全可控，且未观察到新发AEs。然而，有研究^[11]^表明，在免疫联合化疗治疗的患者中，≥3级免疫相关AEs的发生率显著高于免疫单药治疗的患者。此外，Morimoto等^[15]^的研究发现，在75岁及以上接受免疫联合铂类和培美曲塞治疗的患者中，≥3级肺炎的发生率达到16.0%。因此，对于年龄在75岁及以上的患者群体，免疫治疗并非禁忌证，但采用免疫联合化疗方案时应持谨慎态度。为了更好地确保治疗的安全性，建议在开始抗肿瘤治疗前对患者进行全面的老年综合评估。这有助于识别潜在的风险因素，并可能指导个性化的治疗决策，从而优化患者的治疗效果和安全性。

然而，本研究也存在一定的局限性。首先，由于本研究是基于回顾性分析，可能存在选择偏差和信息偏差，这可能影响数据的完整性和准确性。其次，本研究的样本量有限，在进行分析的过程中可能由于样本量不足而导致结果存在一定的偏倚。尽管如此，我们采取了PSM等方法来减少潜在的偏差，并努力确保分析的严谨性。未来仍需要大样本的临床研究进一步验证老年NSCLC患者接受免疫联合化疗治疗的疗效和安全性。

综上所述，本研究在75岁及以上的晚期NSCLC患者中，对比研究了免疫联合化疗和免疫单药治疗疗效及安全性。研究结果表明，匹配前后免疫联合化疗相较于免疫单药治疗，并未在OS或PFS上展现出显著优势，匹配前免疫联合化疗组任意等级AEs发生率较高，匹配后两组安全性相似。这提示临床医生在为老年晚期NSCLC患者制定治疗方案时，应综合评估患者的整体健康状况、疾病特征及潜在的治疗风险，以实现个体化治疗。此外，鉴于当前研究的局限性，未来研究需通过前瞻性、多中心的临床试验来进一步探索免疫治疗在老年NSCLC患者中的最佳应用策略，包括确定预测治疗反应的生物标志物、评估不同联合治疗方案的效果，以及开发针对免疫治疗相关AEs的有效管理策略，以期为75岁及以上的晚期NSCLC患者群体提供更加精准和有效的治疗选择。
